# Assembly of Chitosan/Caragana Fibers to Construct an Underwater Superelastic 2D Layer-Supported 3D Architecture for Rapid Congo Red Removal

**DOI:** 10.3390/nano14181510

**Published:** 2024-09-17

**Authors:** Ning Luo, Hanwen Ge, Xiangyu Liu, Qingdong He, Wenbo Wang, Wenyuan Ma, Fang Guo

**Affiliations:** 1 College of Chemistry and Chemical Engineering, Inner Mongolia University, Hohhot 010021, China; 32307138@mail.imu.edu.cn (N.L.); ghw15001010907@163.com (H.G.); lxy13257244737@163.com (X.L.); 18847744604@163.com (Q.H.); mawenyuaner@163.com (W.M.); 2 SINOPEC Economic & Technical Research Institute Co., Ltd., Beijing 100029, China

**Keywords:** chitosan, caragana, architecture adsorbent, adsorption, dye

## Abstract

Developing environmentally friendly bulk materials capable of easily and thoroughly removing trace amounts of dye pollutants from water to rapidly obtain clean water has always been a goal pursued by researchers. Herein, a green material with a 3D architecture and with strong underwater rebounding and fatigue resistance ability was prepared by means of the assembly of biopolymer chitosan (CS) and natural caraganate fibers (CKFs) under freezing conditions. The CKFs can randomly and uniformly distribute in the lamellar structure formed during the freezing process of CS and CKFs, playing a role similar to that of “steel bars” in concrete, thus providing longitudinal support for the 3D-architecture material. The 2D layers formed by CS and CKFs as the main basic units can provide the material with a higher strength. The 3D-architecture material can bear the compressive force of a weight underwater for multiple cycles, meeting the requirements for water purification. The underwater compression test shows that the 3D-architecture material can quickly rebound to its original shape after removing the stress. This 3D-architecture material can be used to purify dye-containing water. When its dosage is 3 g/L, the material can remove 99.65% of the Congo Red (CR) in a 50 mg/L dye solution. The adsorption performance of the 3D architecture adsorbent for CR removal in actual water samples (i.e., tap water, seawater) is superior than that of commercial activated carbon. Due to its porous block characteristics, this material can be used for the continuous and efficient treatment of wastewater containing trace amounts of CR dye to obtain pure clean water, meaning that it has great potential for the effective purification of dye wastewater.

## 1. Introduction

The continuous advancement of modern industry has promoted economic development, but this has also brought with it severe water pollution problem. The nonbiodegradable organic pollutants (e.g., dyes) discharged from industrial wastewater have caused significant hazards to the ecological environment and even human health [1,2,3]. Dye pollutants are not only highly toxic and nonbiodegradable themselves, but can also prevent sunlight from entering water bodies, affecting the photosynthesis of aquatic plants, resulting in the death of a large number of animals and plants, thereby causing serious harm to ecosystems, even at low concentrations [4].

In recent years, a variety of technologies and methods have been explored to eliminate dye pollutants in water, including Fenton oxidation [5], chemical precipitation [6], membrane filtration technology [7,8], photocatalytic degradation [9,10], adsorption [11,12,13], etc. Among these, the adsorption method has the advantages of simple operation, low cost, high removal efficiency, low energy consumption, easy regulation of the adsorbent structure, and less secondary pollution, etc. It shows great application prospects in the removal of dyes, heavy metals, and other pollutants [14,15]. At present, various adsorbents such as activated carbon [16], carbon nanotubes [17], resins [18], activated diatomaceous earth [19], mesoporous silicate [20], metal–organic framework (MOF) materials [21], and polymer composite materials [22] have been employed to adsorb and remove dye pollutants from polluted water. Due to their more diverse structures and high adsorption efficiency, polymer-based adsorbents have received more attention in recent years [23]. According to the types of pollutants, researchers can design different polymer-based adsorbents such as beads [24], blocks [25], membranes [26], and fibers [27] by changing the types of functional groups (i.e., carboxyl, sulfonic acid groups) or constructing new network structures [28,29]. Bulk adsorbents can better meet the actual use demands because they can be easily separated from the solution.

As “green development” has received more and more attention, the environmental safety of materials has also become the focus of attention. Adsorption materials can not only meet the requirements for usage performance, but also have the advantages of low cost and environmental friendliness [30]. Therefore, the design and development of new types of green adsorbents with superior adsorption ability, safety, environmental friendliness, and low cost have become the focus of widespread attention. Biomass is a green resource derived from nature. It has the inherent advantages of abundance, low price, and safety, meaning that it is suitable for the preparation of environmentally friendly adsorption materials [31]. A variety of natural-polymer-based adsorption materials have been prepared using green methods and renewable resources such as chitosan, chitin, cellulose, alginate, pectin, and lignin, which have been commonly recognized as sustainable and environmentally friendly adsorption materials [32,33,34,35]. However, most of these natural polymers, such as collagen, cellulose, and sodium alginate, are soluble or swellable in water, meaning that the traditional materials derived from them are usually unstable or become swollen underwater, and struggle to return to their original shape [36,37]. These drawbacks limit their application as adsorbents in water [37]. In order to overcome these shortcomings, researchers have exerted much research effort. Si et al. [38] constructed an ordered porous network through the combination of konjac glucomannan and SiO_2_ nanofibers to achieve high elasticity and high mechanical strength. Luo et al. [39] reported a cellulose nanofiber/chitosan composite aerogel with a 3D layered structure, which showed good underwater strength (25.2 kPa under 60% compression strain) and underwater hyperelasticity. Yang et al. [40] proved that bionic structures confer excellent mechanical strength and elasticity on network-like polymer materials. Therefore, designing a stable network structure through the combination of polymers and fibers provides a feasible strategy to improve the mechanical properties of 3D porous polymer materials.

Chitosan ((1,4) -acetylamino-2-deoxy-β-d-glucose) is a linear polysaccharide, a natural polymer obtained via the deacetylation of chitin (e.g., crab, shrimp, or insect shells) [41]. It is naturally nontoxic, environmentally friendly, biocompatible, and biodegradable [42]. In addition, chitosan is rich in amino and hydroxyl groups in its macromolecular chain, which is conducive to the adsorption of various anionic organic pollutants. It is usually insoluble in water and does not swell after molding, meaning that it has the potential to be applied in the field of wastewater treatment [43]. Caragana is a plant that can be grown in arid and semi-arid areas. It is a kind of shrub plant that is widely distributed and cultivated in nature. Caragana branches are stubbled once every 2–3 years, producing a large number of caragana branches [44]. The content of cellulose in the branches of Caragana is high, so natural fibers can be extracted from them. Because the inner core of caragana is hard and firm, its fibers have good toughness and are able to form strong hydrogen bonds and physical entanglement with other polymer chains. Therefore, these fibers can be used as a reinforcing agent to enhance the strength of polymer materials [45,46]. In addition, a large number of hydroxyl groups are present in cellulose, which can be easily modified or functionalized to form adsorption materials with an improved structure and performance [47,48].

In order to develop a novel green adsorbent with excellent performance, environmentally friendly advantages, and superior mechanical strength underwater, natural caragana nanofibers (CKFs) were embedded in a CS matrix through a physical freeze-drying process to construct a CKF/CS 3D-architecture adsorbent. The structure of the material was studied by means of Fourier transform infrared spectroscopy (FTIR), X-ray diffraction (XRD), scanning electron microscopy (SEM), Raman spectroscopy, and X-ray photoelectron spectroscopy (XPS) analyses, and the mechanical properties and adsorption performance were evaluated using Congo Red (CR) dye as a model pollutant. This study is expected to provide theoretical and technical support for the design of high-strength and high-efficiency green adsorption materials.

## 2. Experimental Section

### 2.1. Materials and Methods

Caragana branches were taken from Siziwang Banner in the Inner Mongolia Autonomous Region, provided by Inner Mongolia Menglin Ecological Technology Co., Ltd. (Inner Mongolia, China). Chitosan (CS) (degree of deacetylation: ≥95%) was purchased from Shanghai Maclin Biochemical Technology Co., Ltd. (Shanghai, China) (structure formula is shown in Figure S1) [49]. Congo Red (CR) was purchased from Alfa Esha (China) Chemical Co., Ltd., Shanghai, China (see Figure S2). Acetic acid was purchased from Tianjin Fengchuan Chemical Reagent Co., Ltd. (Tianjin, China). Sodium hydroxide was purchased from Aladdin reagent Co. (Shanghai, China). Activated carbon 1 (named CAC1) was purchased from Thermo Fisher Waltham, MA, USA. Activated carbon 2 (named CAC2) was purchased from Sinopod Chemical Reagent Co., Ltd., Shanghai, China. Activated carbon 3 (named CAC3) was purchased from Alfa Esha (China) Chemical Co., Ltd.

### 2.2. Preparation of Caragana Fiber

The caragana branches were cut into pieces and then ground into powder using a high-speed grinder. The pretreated caragana powder (8 g) was added to a 4 wt.% NaClO_2_ solution at pH 4.6, and the mixture was stirred mechanically at 80 °C and 500 rpm for 8 h. The treated samples were filtered through a Brinell funnel and then washed with deionized water several times. The washed product was further reacted with an 8 wt.% NaOH solution at 80 °C for 4 h and then filtered through a Brinell funnel to obtain aggregates of fibers. The product was then washed fully with deionized water until the pH was close to neutral. The fibers were dispersed in an appropriate amount of deionized water, homogenized at 15,000 rpm for 5 min using a high-speed homogenizer, and then further dispersed by means of an ultrasonic treatment using a cell crusher for 10 min to obtain a uniform suspension of fibers. The solid in the aqueous suspension was filtered out and dried in an oven at 60 °C for 24 h to obtain caragana fibers. The TEM image of caragana fibers is shown in Figure S3. The nanosized fibers can be clearly observed.

### 2.3. Preparation of Chitosan/Caragana Fiber Composite Materials

The caragana fibers (0.1 g, 0.15 g, 0.2 g, 0.5 g, and 0.3 g, respectively) (Figure S4) were dispersed in 98 mL of water in a beaker by employing a high-speed homogenizer (Xicheng Xingri Instrument Factory, Jintan District, China) to obtain a uniform aqueous suspension. Subsequently, chitosan (2 g) was added into the suspension, and the resultant mixture was treated with ultrasonication for 5 min using an ultrasonic cell pulverizer, followed by even stirring at 600 rpm. During the mechanical stirring process, 2 mL of acetic acid was slowly added, and the mixture was stirred continuously for 4 h. The obtained mixture was poured into a mold, left to stand for 30 min, and then frozen at −80 °C for 12 h and vacuum-dried for 48 h with a vacuum freeze dryer (LGJ-12) (Beijing Songyuan Huaxing Technology Development Co., Ltd., Beijing, China) to obtain chitosan/caragana fiber products. The products were treated with a 2 mol/L NaOH solution and then washed with deionized water until the pH became neutral. The washed product was frozen at −80 °C for 12 h and vacuum-dried for 48 h to obtain the final product. The 3D-architecture materials with different addition amounts of CKFs were prepared. It was shown that the architecture material prepared by adding 0.2 g of CKFs had the best adsorption capacity (Figure S5). The optimal chitosan/caragana fiber composite was labeled as CS/CKF. The CS monolith was also prepared under the same condition for comparison purposes.

### 2.4. Resilience and Compression Cycle Test

The CS/CKF composite material was compressed in air and underwater pressure tests using a 500 g weight. The dried composite material was placed in a beaker and a moderate amount of deionized water was added until the liquid level submerged the composite material. The weight was placed on the composite material in air and underwater, respectively, and the resilience and shape of the composite material were observed. The resilience of the CS-based composite material and the CS/CKF composite material was compared, the resilience time was recorded, and the fatigue resistance of CS/CKF was investigated.

### 2.5. Stress–Strain Test

Stress and strain tests of the CS/CKF composite material in air and underwater were performed using a material testing machine. The stress variable of the air pressure test was set as 50% and 80%, while the stress variable of compression underwater was set as 70%, so as to explore the change in stress under different strain conditions.

### 2.6. Dye Adsorption Experiment

The CS/CKF material (0.0100 g) was placed in 20 mL of CR solution, and the mixture was oscillated in an oscillating chamber at 30 °C for 4 h. Then, the adsorbent was separated from the CR solution by a filtration membrane. The absorbance of the CR solution before and after adsorption was measured with a UV–visible spectrophotometer, and then the concentration of CR in the solution was determined via the standard curve method. The adsorption amount of CR per unit mass of CS/CKF (*q*_e_, mg/g) was calculated according to Equation (1) [50].

*q*_e_ = [(*C*_0_ − *C*_e_) × *V*]/*m* (1)

In this equation, *C*_0_ is the initial concentration of the CR solution (mg/L); *C*_e_ is the concentration of the CR solution at adsorption equilibrium (mg/L); *V* is the volume (L) of the CR solution used in the adsorption experiment; and *m* is the mass of the adsorbent (g). The influence of the initial pH on the adsorption performance was studied. The initial concentration of the CR solution was 200 mg/L, and the pH value of the CR solution was adjusted from 5 to 12 to evaluate the influence of the external pH value on adsorption.

### 2.7. Test of Adsorption Efficiency of Adsorbent

The 3D-architecture material was used to adsorb CR dye in solutions with initial concentrations of 50 mg/L and 10 mg/L (adsorbent dosage: 0.5 g/L, 1.0 g/L, 2.0 g/L, 3.0 g/L and 4.0 g/L, respectively), and the removal rates of dye at the two concentrations were investigated. The adsorption materials (0.01 g, 0.02 g, 0.04 g, 0.06 g and 0.08 g, respectively) were added to 20 mL of the dye solution, and then the mixture was shaken at 120 rpm and 30 °C for a certain time in an oscillating chamber. After the adsorption reached equilibrium, the adsorption material was separated from the solution by means of a filtration process. The UV–visible spectra of the dye solution were scanned, and digital photos of the solution were taken.

The dynamic adsorption efficiency of the adsorbed materials was tested by means of the following procedure. A simple peristaltic pump adsorption device was installed, and a single-architecture material (mass of ~0.2 g) was installed on the outlet end of the peristaltic pump catheter. Under the action of the peristaltic pump, the CR solution (5 mg/L) entered from one end of the pump tube at a flow speed of 5 mL/min, and then flowed through the material for adsorption. The concentration of dye in the effluent and the original solution was detected, and the UV–visible spectra of each solution were scanned.

The adsorption material (dosage: 3 g/L) was used to adsorb CR dye in different water samples (e.g., ultrapure water, tap water, seawater) (dye concentration: 50 mg/L). Meanwhile, the adsorption efficiency of three commercial activated carbons (i.e., CAC1, CAC2, CAC3) was tested. The adsorption performance of the architecture material was compared with CS/CKF. The removal efficiency of the CR dye by four kinds of adsorbents (dosage: 3 g/L) in actual water (i.e., tap water, sea water) was also investigated. The UV–visible spectra of the CR solution before and after adsorption were obtained using a UV–visible spectrophotometer, and digital photos of the solution before and after adsorption with different amounts of adsorbent were taken.

### 2.8. Characterizations

The mechanical properties of CS/CKF were tested using the Universal Material Tester (AGS-X, Shimadzu, Kyoto, Japan). The concentration of dye solution was measured with a UV–visible spectrophotometer (UV1900i, Shimadzu Instrument Co., Ltd., Kyoto, Japan). The surface morphology of the sample was observed with a scanning electron microscope (ZEISS SUPRA55, Carl Zeiss, Oberkochen, Germany); the sample was glued to a conductive adhesive and then sprayed with gold. The morphology of the fibers was observed with a transmission electron microscope (TEM, FEI Tecnai F20 S-Twin, 200 kV) equipped with a high-angle annular dark-field detector (HADDF) and an energy-dispersive spectrometer (EDS). The composition of elements on the surface of the sample was investigated using an X-ray photoelectron spectrometer (Thermo Fisher, Waltham, MA, USA), and high-resolution spectrograms of the C 1s, N 1s, O 1s, and P 2p regions were obtained. The infrared spectra of the sample were obtained with the Nicolet IS 10 infrared spectrometer (Thermo Fisher, Waltham, MA, USA) in a wavenumber range of 4000–400 cm^−1^, and the sample was pressed with KBr. The crystal phase composition of the sample was established using an X-ray powder diffractometer (X ‘PERT PRO, Bruker, Karlsruhe, Germany). The Raman spectra were obtained with a Raman spectrometer (LabRAM HR Evolution, HORIBA, Longjumeau, France), focusing on the D and G bands.

## 3. Results and Discussion

### 3.1. Structural and Morphology Analysis of Monolith

**Figure 1:**
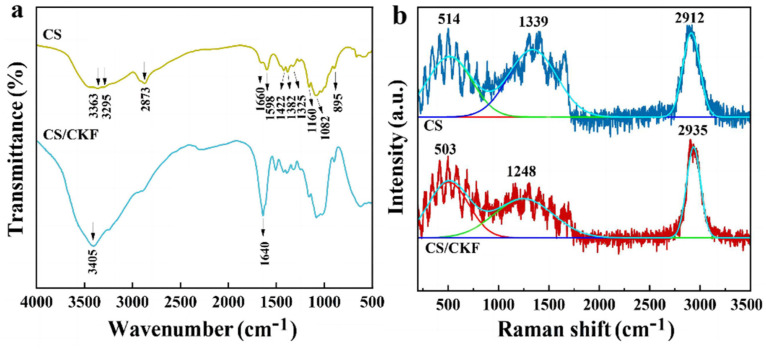
(**a**) FTIR spectra of CS and CS/CKF; (**b**) Raman spectra of CS and CS/CKF.

The structure of CS/CKF was analyzed via FTIR spectroscopy, Raman spectra, and XPS. Figure 1a shows the FTIR spectra of the CS and CS/CKF composites. The main characteristic bands of CS mainly appear at 3363 cm^−1^ (O–H stretching vibration), 3295 cm^−1^ (N–H stretching vibration), and 2873 cm^−1^ (CH stretching vibration), which indicates the presence of amino (–NH_2_), hydroxyl (–OH), –CH_2_- and –CH_3_ groups, respectively [51,52]. The bands at 1660 cm^−1^ and 1598 cm^−1^ are related to the amide I band (C=O stretching vibration) and the amide II band (N–H bending vibration), respectively [53,54]. The bands at 1382 cm^−1^ and 1325 cm^−1^ correspond to the symmetric stretching vibration of amido-CH_3_ and the stretching vibration of the pyrane ring (CH/CH_2_), respectively, which are ascribed to the amide III band [55,56]. The small peak at 895 cm^−1^ is ascribed to the beta bond of the glucoside ring [57]. The peaks at 1082 cm^−1^, 1030 cm^−1^, and 1160 cm^−1^ belong to the C–C and C–O stretching vibrations, which are characteristic peaks of polysaccharides [55,58,59]. For the CS/CKF composite, the sharp absorption bands appear at 3405 cm^−1^ (–OH stretching vibration) and 3295 cm^−1^ (the N–H stretching vibration peak of -NH_2_), indicating that abundant levels of –OH and –NH_2_ are present in CS/CKF. The characteristic band at 1640 cm^−1^ overlaps with the peak of the amide II band at 1598 cm^−1^, which is related to the hydrogen bonding interactions between the –NH_2_ and –OH groups, indicating that the CKFs and CS are combined through hydrogen bonding interactions (Figure S6) [60,61]. In the FTIR of CKFs, the main characteristic bands appear at 3412.9 cm^−1^ (–OH stretching vibration), 2914.4 cm^−1^(C–H stretching vibration), 1635 cm^−1^ (C=C stretching vibration), 1053.5 cm^−1^ (C–O stretching vibration), 588.3 cm^−1^ (C–H stretching vibration). These results indicate that hydroxyl, methyl, and amide groups existed. In the CS/CKF composite, a sharp absorption band appears at 3363 cm^−1^(–OH stretching vibration), indicating that the hydroxyl group in CS/CKF composite is not only derived from chitosan, but also from caragana fibers. The hydroxyl group in caragana fibers is bound to the amino group of chitosan through hydrogen bonding interactions (Figure S7). Chitosan may bind to anionic dye via the protonation of the amino group, and therefore the mass ratio of CS to CKFs is especially important. With the increase in CKF content, the number of amino groups tends to be reduced. Therefore, it is particularly important to optimize the ratio of the 3D-architecture material. It has been proven that the CS/CKF0.2 composite has the best adsorption performance, because it contains both -NH_2_ and -OH as the active adsorption sites.

The Raman spectra of CS and CS/CKF are shown in Figure 1b. The basic structural unit of a CS molecule is glucose, which simultaneously contains an amino group, an acetyl group, and a hydroxyl group [62]. The characteristic peak at 2912 cm^−1^ is attributed to the C-H stretching vibration in the polysaccharide skeleton, the absorption peak at 1339 cm^−1^ is ascribed to –CH_2_ and –CH_2_OH, and the absorption peak at 514 cm^−1^ is attributed to the exocyclic vibration of the sugar ring [63,64,65]. From the Raman spectra of CS and CS/CKF, it can be found that the Raman spectra of CS did not change significantly after forming the 3D-architecture material, and only a slight shift in peaks was observed. This indicates that hydrogen bonding is the main interaction between polymer chains, and no chemical reaction occurred between groups (i.e., amino and hydroxyl groups).

**Figure 2:**
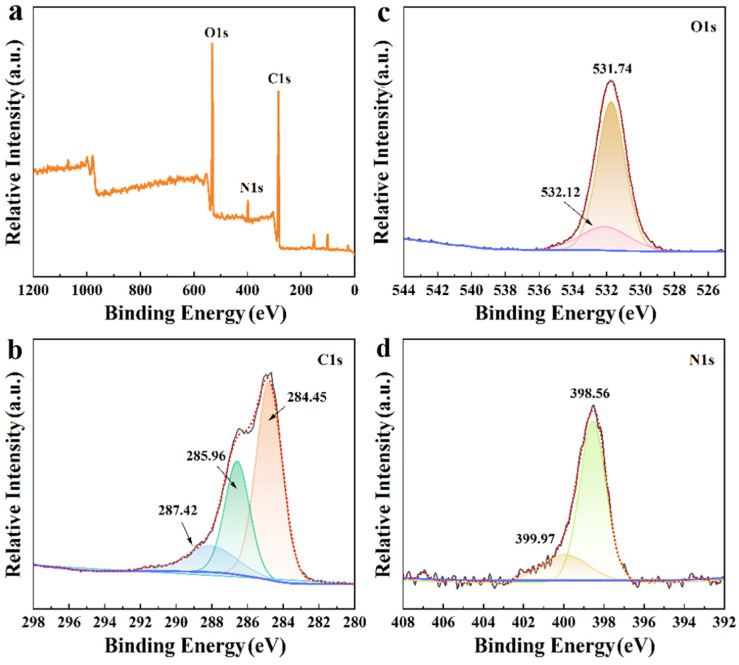
Survey scanning XPS spectra of CS/CKF (**a**) and fine scanning XPS spectra of C 1s (**b**), O 1s (**c**), and N 1s (**d**) of CS/CKF.

In order to explore the structure and chemical composition of CS/CKF, the XPS spectra of CS/CKF were analyzed. The signal peaks of O 1s, N 1s, and C 1s can be observed in Figure 2a, which represent the main elements in the CS/CKF composite material. The C 1s signal peak (Figure 2b) can be split into three peaks at binding energies of 287.42 eV (COO–H), 285.96 eV (C–OH), and 284.45 eV (C–C), respectively. This indicates that there are abundant C–OH groups and –COOH groups on the surface of CS/CKF [66,67]. The presence of these groups can also be proven by the O 1s spectral analysis. The O 1s peak can be split into two peaks at 531.12 eV (C=O) and 531.74 eV (–OH) (Figure 2c) [68,69]. In addition, the N-containing functional groups on CS/CKF were analyzed. The N 1s peak can be split into two peaks at 399.97 eV (–NH_2_) and 398.56 eV (C–N) (Figure 2d), indicating that amino groups are present on CS/CKF [70]. These results indicate that the CS does not chemically react with CKFs to form new chemical bonds, and CKFs and CS are held together by hydrogen bonding. Compared with the adsorption of Congo Red dye by CKFs or CS, it was seen that the adsorption effect of caragana fibers on Congo Red dye is not obvious, and chitosan plays a major role in the adsorption of Congo Red onto this material (Figure S8).

**Figure 3:**
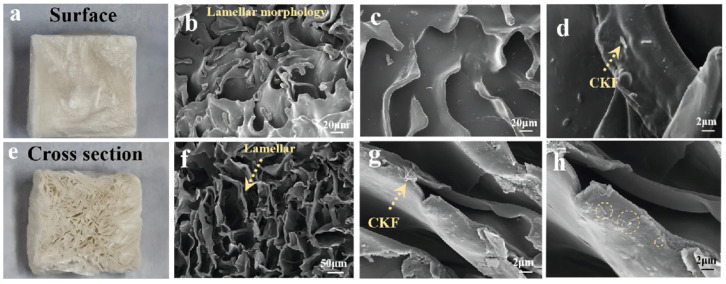
Digital photographs of surface (**a**) and cross-section (**e**) of CS/CKF; SEM images of the surface (**b**–**d**) and cross-section (**f**–**h**) of CS/CKF at different magnifications; the dashed circles in subfigure h represents the fiber in polymer substrate.

The surface morphology of the outer surface and cross-section of CS/CKF was observed (Figure 3). As shown in Figure 3a, the outer surface of the 3D-architecture material presents a cross-overlapping layered stacking structure, and there are a large number of pores between layers. As can be seen from Figure 3b, many CKFs are randomly distributed and attached to it. The CS polymer was combined with CKFs to form a layered structure, and these layers supported the structure of the CS/CKF 3D-architecture material. As shown in Figure 3c, the cross-section of CS/CKF exhibits many irregular porous structures, which are composed of pores stacked with lamellar structures on their surface. The hole wall thickness is about 5 μm (Figure 3d). Meanwhile, the SEM image of the cross-section shows that CKFs are not only attached to the surface of the lamel, but also partially embedded in the lamel. The length of the CKFs is about 2 μm, and they are randomly distributed between the pore wall and the lamel in short cylindrical rods. Since cellulose can form both intramolecular and intermolecular hydrogen bonds, intramolecular hydrogen bonds will limit the rotation of the glycoside bond, leading to a great increase in its rigidity. Therefore, the uniform distribution of CKFs in CS/CKF is beneficial to improve this composite’s mechanical properties. In the transversely stacked lamellar structure, CKFs provide a kind of longitudinal support, providing this composite with a strong ability to resist damage induced by external force during the compression process.

### 3.2. Mechanical Property

**Figure 4:**
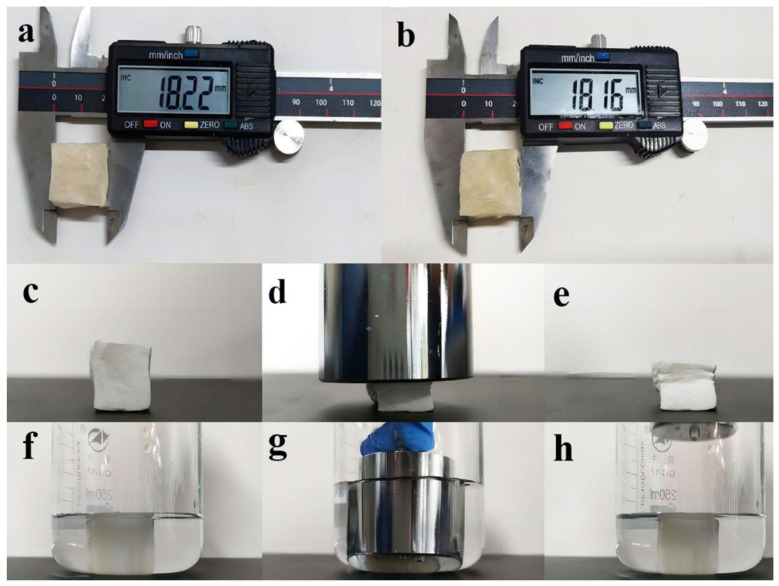
Height comparison photos of CS/CKF before (**a**) and after (**b**) 100 repetitions of compression; the photographs of CS/CKF in the air (**c**–**e**) and underwater (**f**–**h**) pressure tests.

Three-dimensional-architecture materials with better mechanical properties are more suitable for removing pollutants from water. Therefore, the underwater pressure test and the air pressure test were carried out to examine the mechanical properties of the CS/CKF composite. As shown in Figure 4c–h, a pressure test in the air was conducted by adding a 500 g weight onto the material (Figure 4c–e). A downward force of 10 N was applied to compress the upper surface of the material, and then the weight was removed. It was found that the composite material deformed and had a small range of rebound after compression. The same amount of force was applied to the composite underwater using the same weight (Figure 4f–h). After the weight was removed, the 3D-architecture material bounced back rapidly with almost no deformation compared to its original shape (Video S1). This observation shows that the 3D-architecture material exhibits excellent mechanical strength and rebound ability underwater.

In order to further explore the underwater fatigue resistance of the CS/CKF material, the cyclic compression test was carried out underwater. The fatigue resistance was evaluated by repeating the compression cycle test with a weight. After the material was compressed with the weight, the weight was removed immediately. After 100 compression–release cycles, the size of the material was measured with a vernier caliper. The size of material changed from 18.22 mm (before compression) to 18.16 mm (after compression) (Figure 4a,b). These results show that the CS/CKF material has high resilience and compression resistance underwater and has good fatigue resistance.

**Figure 5:**
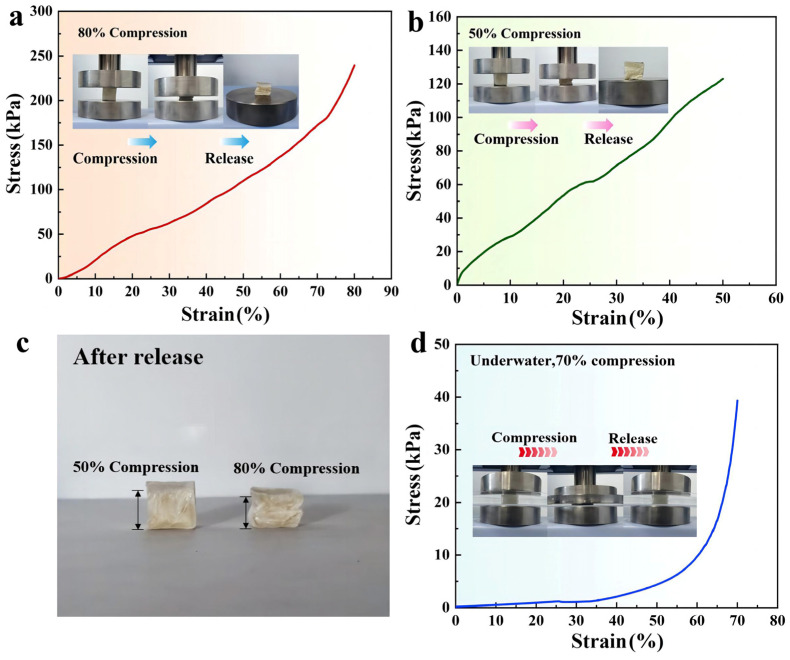
Compressive stress versus strain curves for the CS/CKF in air at 80% strain (**a**) and 50% strain (**b**); recovery of CS/CKF after release in the 50% and 80% strain tests (**c**); compressive stress versus strain curves for the CS/CKF underwater at 70% strain (**d**).

In order to study the mechanical properties of the CS/CKF material in greater depth, stress and strain tests were carried out in air and underwater. The different stress–strain curves of the material in air at strains of 50% and 80% are plotted in Figure 5a,b. Due to the existence of a tight spring-like layered structure inside the CS/CKF material (Figure 3f), it has superior elastic characteristics at a strain below 70% (Figure 5b,c). With the further increase in strain, the lamellar structure compresses gradually, and the lamellar structure inside the 3D-architecture material gradually stacks together, resulting in a rapid increase in the compressive stress at 75% strain. For the architecture material with a 50% stress–strain test, it recovered to 90% of its original height within 60 s after unloading. When the material underwent an 80% stress–strain test, about 80% of its original height was recovered within 60 s after unloading (Figure 5c). Compared with the underwater compression performance of the material, the rebound rate of the material in air is much slower, and the shape of the material cannot be recovered fully.

Figure 5d shows the stress–strain curve of CS/CKF underwater at a strain of 70%. When CS/CKF material is compressed under 70% strain, its maximum compressive stress reaches 49.0 kPa. When the strain variable is less than 25%, the force behavior of the material basically conforms to Hooke’s law, the stress and the strain become proportional, and then the lamellar structure of the material continues to stack. When the strain reaches 60%, a small increase in strain can cause a rapid increase in compressive stress. The stress–strain curve of CS/CKF underwater is smoother than that in the air and is more in line with the characteristics of elastic deformation. The CS/CKF material can rapidly rebound after being pressed (Video S2) because the pores between the inner layers are filled with water when the material is underwater. When the material is compressed by an external force, the water in the pores will be squeezed out. When the force is released, the pores between the inner layers of the CS/CKF material are filled with water instantly, and the layered stacking structure is restored to its original state instantly, meaning that the CS/CKF material exhibits more stable properties underwater. At the same time, due to the good toughness and high strength of the CS/CKF material, its lamellar structure will not damage its internal microstructure during extrusion, so the material can almost bounce back completely after deformation.

### 3.3. Adsorption Properties

**Figure 6:**
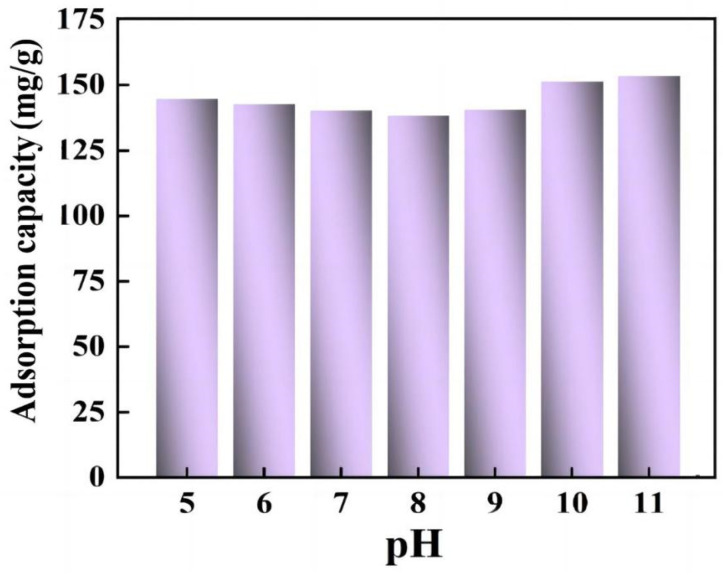
Effect of pH on the adsorption of CR by the CS/CKF material.

The initial pH value of the solution may affect the presence of dye and the surface properties of the adsorbent, and thereby affect the adsorption behavior of the adsorbent. The adsorption behavior of the CS/CKF material regarding CR dye was studied in a pH range of 5~12. Since CR dyes are unstable under acidic conditions, the pH values of the CR solution were controlled at ≥5. As shown in Figure 6, with pH values ranging from 5 to 11, the adsorption efficiency of CS/CKF does not change significantly, indicating that the material has good pH stability. Due to the presence of –NH_2_ and –OH groups in the polymer chain, CS serves as a coordination and electrostatic interaction site for the adsorption of anionic dyes. The –OH and –COOH groups in the material can bond with anionic dyes through hydrogen bonding interactions. The SO_3_Na groups in the CR molecules can form strong interactions with the –NH_2_ groups in the CS/CKF material to promote the adsorption of CR. The CKFs containing large numbers of –OH and –COOH groups can also contribute to the adsorption of CR through forming hydrogen bonding interactions with the dye.

**Figure 7:**
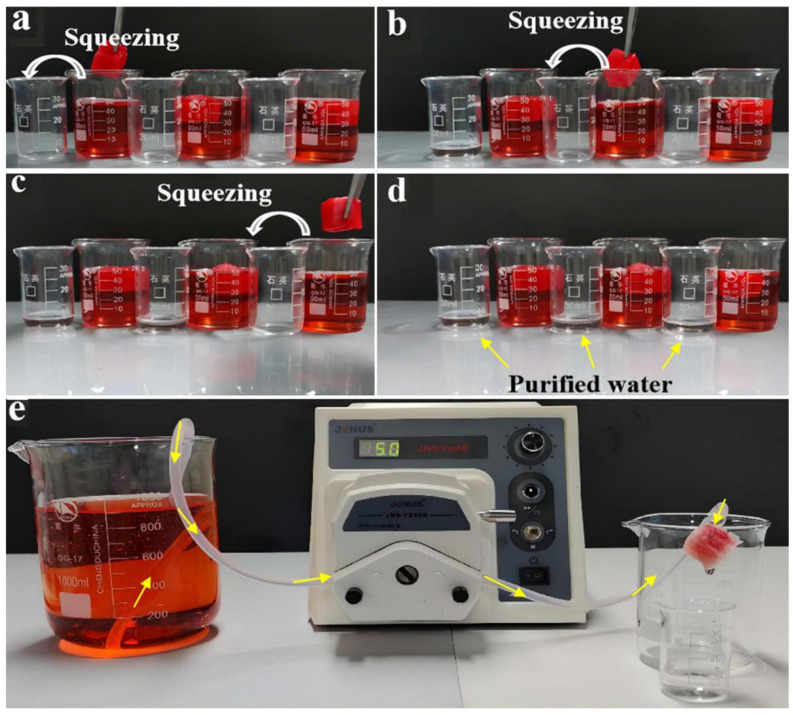
(**a**–**d**) Photographs illustrating the facile removal of CR using the CS/CKF; (**e**) photographs showing the CR separation process by in situ pumping in 5 mL/min.

The underwater superelasticity, high resilience, and fatigue resistance of the CS/CKF material make it suitable for use in the dynamic purification of wastewater. As shown in Figure 7a–d, the 3D-architecture material was compressed to a deformation greater than 80%, and then it was put into a red CR solution (50 mg/L) to test its adsorption of CR in an aqueous solution. The compressed material quickly returned to its original shape after coming into contact with the CR solution. It is encouraging that when the water-saturated material was picked up with tweezers and squeezed again, the extruded water became colorless and transparent, and the material turned red, indicating that the material captured the CR in the water and released clean water. In addition, the CS/CKF material can be used as a filter to purify CR-containing water via a continuous and convenient peristaltic pumping method. As shown in Figure 7e, the peristaltic pump can drive the CR solution (5 mg/L) to flow freely through the adsorption material at a peristaltic pump speed of 5 mL/min, and transparent and colorless water was collected at the other end of the peristaltic pump output (Video S3), thus achieving fast and efficient water purification. The UV–visible spectra of the solution before and after purification were scanned to evaluate the adsorption effect. It was found that the characteristic absorption peak of CR disappeared after purification with the 3D-architecture material, and the removal efficiency of CR was calculated to be 99.27%. At the same time, we performed the adsorption isotherm experiment of Congo Red on the material. When the initial concentration of dye was 200 mg/L, the maximum adsorption capacity of Congo Red on the material was 132.56 mg/g (Figure S9a). In addition, the Langmuir model and Freundlich model were used to fit the adsorption data, and the *R*^2^ value obtained by the Langmuir model was greater than 0.99. The adsorption amount of CR calculated according to the Langmuir isotherm model was consistent with the experimental value. The results show that the adsorption process of Congo Red dye was consistent with Langmuir isothermal model, and the adsorption process consisted of single-molecular-layer adsorption (Figure S9b, Table S1).

**Figure 8:**
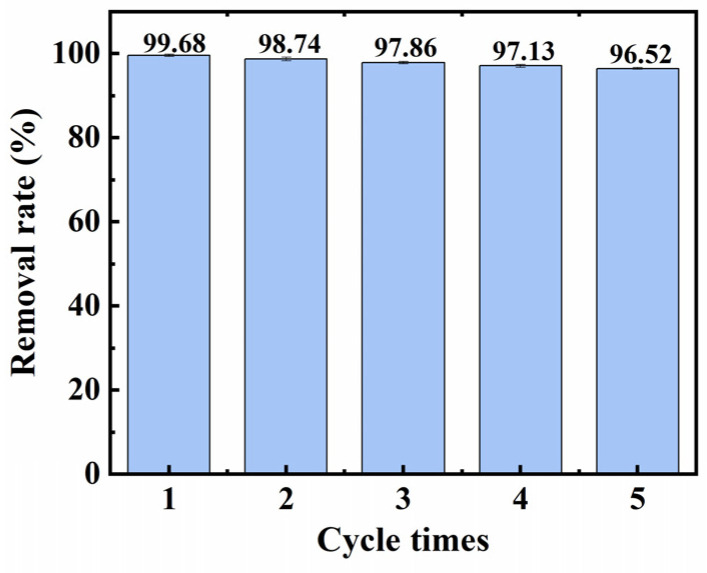
Cyclic adsorption experiment of the adsorption material.

As an important parameter for adsorbents [71], the reusability of the composite material was studied through five CR adsorption–resolution cycles. The material was placed in 20 mL of CR solution with a concentration of 200 mg/L, oscillated in a oscillator, and the concentration of CR in the solution was determined after adsorption equilibrium was reached. Then, the material was removed from the CR solution by means of a filtration method, eluted with 0.1 mol/L NaOH and 250 mL of a C_2_H_5_OH solution, washed with deionized water, freeze-dried, and the experiment was repeated. Figure 8 shows the data collected during the experiment. After repeating the adsorption–resolution cycle five times, the adsorption material still successfully removed nearly 96.52% of the CR. These results show that the adsorption material has excellent performance in repeated use, and after many adsorption experiments, the adsorption of CR still demonstrated good performance.

**Figure 9:**
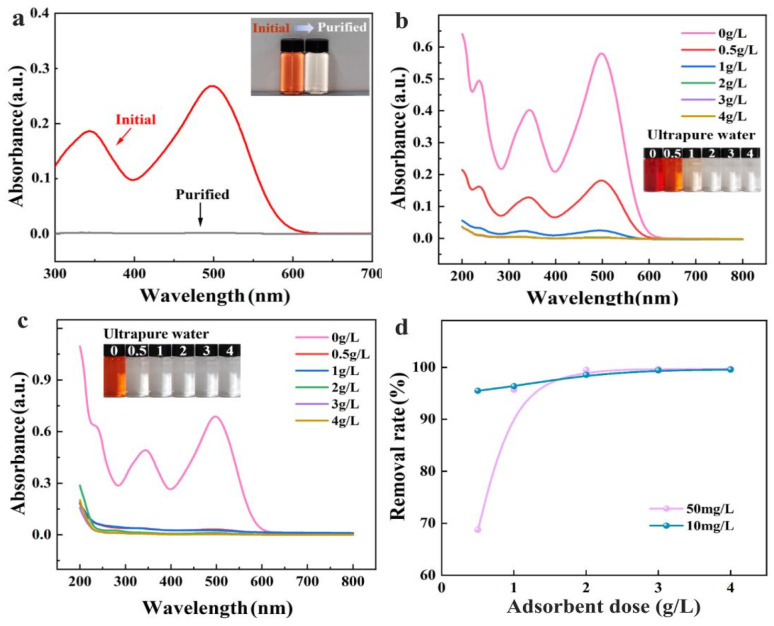
(**a**) UV–visible absorption spectra of the initial CR solution (5 mg/L) and the purified solution after the first separation process. The inset images are photographs of the corresponding solutions; (**b**) UV–visible spectra and digital photos of the CR solution (initial concentration: 50 mg/L) before and after adsorption with different amounts of the CS/CKF monolith; (**c**) UV–visible spectra and digital photos of the CR solution (initial concentration: 10 mg/L) before and after adsorption with different dosages of the CS/CKF monolith; (**d**) the removal rate of different concentrations of CR dye after adsorption with the CS/CKF monolith adsorbent.

In practical applications, it is challenging to remove low-concentration pollutants in actual water bodies. Therefore, the removal efficiency of CS/CKF for low concentrations of CR dye (50 mg/L and 10 mg/L, respectively) was investigated at different dosages of adsorbents (0.5, 1, 2, 3, and 4 g/L, respectively). After adsorption, the UV–visible spectra of the CR solution were scanned and digital photos of the solution were taken to observe the change in color of the dye solution (Figure 9a,b). With the increase in the adsorbent dosage, the absorbance of the CR solution at the maximum absorption wavelength (497 nm) gradually decreased, and the solution gradually lost its color, indicating that the adsorption material is very effective in removing CR from water. When the dosage of the adsorbent material is 2 g/L, CR solution (concentration: 50 mg/L) becomes colorless after adsorption, with a removal rate of 99.48%. When the adsorbent dosage is 3 g/L, the removal rate reaches 99.65%, and when the dosage is increased to 4 g/L, the removal rate remains unchanged. The low-concentration CR solution (initial concentration: 10 mg/L) becomes colorless after adsorption with a 5 g/L dose of the adsorbent, with a high CR removal rate of 95.49%. When the adsorbent dosage is 4 g/L, the removal rate of CR dye reaches 99.56%. After adsorption, the absorbance of the solution at the maximum absorption wavelength in the UV–visible spectrum is close to zero. These results show that the CS/CKF material has strong capture ability for CR in water, even at low concentrations, making it promising for use in the thorough purification of dye wastewater.

**Figure 10:**
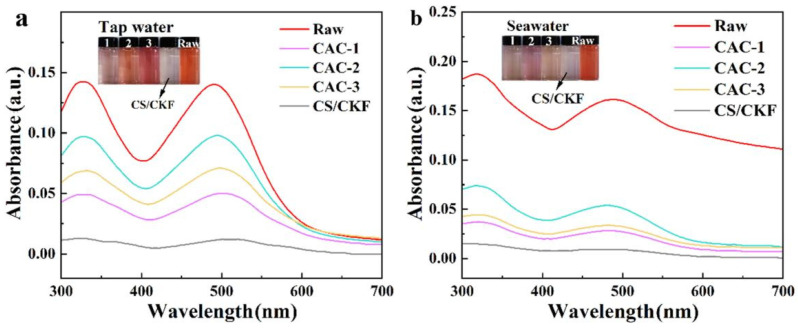
UV–visible spectra of a CR solution in tap water (**a**) and seawater (**b**) before and after adsorption with the CS/CKF material and three types of commercial activated carbon. The inset images are digital photos of the CR solution before and after adsorption.

Because actual water bodies are much more complex than the pure water used in the laboratory, it is very important to discuss the adsorption ability of the adsorbent in actual water conditions, such as tap water, seawater, etc. As shown in Figure 10, it was clearly noticed that the adsorption capacity of CS/CKF material for CR in tap water and seawater is much stronger than that of three commercial activated carbons. In tap water, the CS/CKF material has the best adsorption efficiency for CR in the solution, followed by CAC-1, CAC-3, and CAC-2. The removal rate of the CS/CKF adsorbent for CR in tap water was 91.37%. After adsorption with the CS/CKF material, the solution becomes clear and transparent. The CR solutions adsorbed by the three activated carbons still display different shades of red. In seawater, the CS/CKF adsorbent has the best adsorption effect on the CR solution, followed by CAC-1, CAC-3, and CAC-2. The removal rate of CR in seawater by the CS/CKF adsorbent is 94.41%.

CR is an anionic dye, and the surface negative charge of CR molecules causes them to mutually repel each other to maintain stability [72]. When a certain amount of positively charged electrolyte is added to the CR solution at this time, it reduces the repulsive force between the particles and leads to the flocculation phenomenon.

Over the course of the experiment, it was found that the CR solution would flocculate in seawater and tap water in the absence of an adsorbent, and the flocculation phenomenon in CR solutions is most obvious in seawater. It was found that the conductivity of seawater used in this experiment was 31.29 mS/cm, indicating that there are a lot of free ions and various elements in seawater, such as Na^+^ ions, K^+^ ions, Ca^2+^ ions, Mg^2+^ ions, Cl^−^ ions, SO_4_^2−^ ions, and CO_3_^2−^ ions. The presence of these cations and trace elements may cause the flocculation of CR. Han et al. [73] proposed a method for the efficient removal of the harmful azo dye CR in simulated wastewater via metal ion chelation flocculation and flotation separation. It was shown that CR chelates with trivalent metal ions, including Al(III) and Fe(III), and their mixtures can form hydrophobic flocs. Therefore, the flocculation phenomenon in CR solutions in seawater may be due to the interaction between positively charged ions in seawater and the negatively charged CR molecules, which reduced the thickness of the double electric layer and the repulsive force between particles, resulting in a flocculation phenomenon. The flocculation phenomenon in tap water is not as obvious, because the conductivity of tap water is 80.26 μS/cm, which is far lower than that of seawater. In addition, the ions in the solution may compete with the adsorption sites, leading to a decrease in removal efficiency. However, according to the experimental results, the removal efficiency of the CS/CKF material for CR is still the best compared to three commercial activated carbons. In other words, the CS/CKF material has greater potential for the removal of low-concentration CR in actual water.

### 3.4. Cost–Benefit Evaluation

The cost–benefit evaluation was carried out according to the price of raw materials on the market. As shown in Table 1, the main raw materials used for the preparation of the CS/CKF monolith are chitosan and caragana fibers. Caragana has huge reserves in nature, so its cost advantage is obvious. The price of CS/CKF material is around CNY 1945/t, which is obviously lower than the average market price of commercial activated carbon (CNY 5500/t) for the treatment of dye wastewater. At the same time, 3D-architecture materials are more advantageous for the removal of dyes in actual wastewater. Therefore, the CS/CKF composite has good adsorption properties, mechanical properties, and economic benefits, and has great potential in the actual large-scale purification of dye-polluted water.

## 4. Conclusions

The CS/CKF material with high elasticity, high recovery, and fatigue resistance underwater was prepared through the combination of CS and CKFs. CKFs can randomly and evenly distribute in the CS matrix to support the network structure, resulting in a higher mechanical strength. The material can rapidly rebound during underwater compression tests. The 50% strain test of the CS/CKF material in air shows that it recovers 95% of its original height within 30 s after unloading, while it recovers 80% of its original height 60 s after unloading during the 80% strain test. The CS/CKF material bounces back quickly after stress unloading during the 70% strain test underwater. When the adsorbent dosage is 3 g/L, the removal rate of CR reaches 99.65% after adsorption. At an initial concentration of 10 mg/L and an adsorbent dosage of 4 g/L, the removal rate of CR can reach 99.56%. The 3D-architecture material can be continuously and efficiently used to treat CR-polluted water by driving with a peristaltic pump to obtain clean water, with a removal rate of up to 99.27% for a low-concentration CR solution (5 mg/L). The adsorption material also shows better adsorption capacity for CR in actual water (tap water and seawater) than three commercial activated carbons. Although the adsorption capability of the material for CR in actual water is slightly lower than that in pure water, the removal efficiency can still reach 94.41% for CR in seawater, which is also better than those of commercial activated carbons. In a word, the 3D-architecture material with an excellent adsorption efficiency, low cost, and eco-friendly advantages has the potential to be used for the purification of water contaminated with low concentrations of dye pollutants.

## Figures and Tables

**Table 1:** Cost–benefit analysis of the adsorbents in China.

Project/Operation	Cost/Benefit Parameters	Records
Caragana	CNY 0	Natural biomass
Chitosan	CNY 1000/t	Purchased from the manufacturer
The cost of transporting one load of raw materials	CNY 200–300	Caragana is loaded and transported by truck
Other chemical reagent prices	CNY 300/t	Average market price
Water consumption per batch	10 t water/t	Industrial water price is CNY 3.0/t
Electric energy loss	200 kWh/t	Industrial electricity is priced at CNY 0.725/kWh
Purification capacity of dye wastewater	2 g/L	This work
Total cost	1945 ¥/t	

## Data Availability

The data presented in this study are available upon request from the corresponding author.

## Supplementary Materials

The following supporting information can be downloaded at: https://www.mdpi.com/article/10.3390/nano14181510/s1. Figure S1. The structure formula of chitosan; Figure S2. The structure formula of Congo Red; Figure S3. TEM image of caragana fibers; Figure S4. Images of CS and CKFs before assembling; Figure S5. Effect of the CKF addition amount on adsorption capacity; Figure S6. Hydrogen bonding interaction between the hydroxyl group of CKFs and the amino group of CS; Figure S7 FTIR spectra of CKFs; Figure S8. Adsorption capacity of Congo Red dye displayed by CKFs, CS, and CKF/CS, respectively. Figure S9. Curves of adsorption capacity versus equilibrium concentration (Ce, mg/L) for the adsorption of CR onto the CS/CKF composite material; the plots of Ce/qe versus Ce for the adsorption of CR on the CS/CKF composite material (fitting with Langmuir model); and the plots of log qe versus log Ce for the adsorption of CR on the CS/CKF composite material (fitting with Freundlich model). Video S1. Video of the rebound behavior of the monolith after loading and releasing weights. Video S2. The rebound behavior of the monolith underwater after repeating the press–release cycle. Video S3. Video depicting the continuous kinetic purification of CR-containing water. Table S1. Fitting parameters with Langmuir model and Freundlich model for adsorption of CR.
