# Supplementary material for: Assembly of Chitosan/Caragana Fibers to Construct an Underwater Superelastic 2D Layer-Supported 3D Architecture for Rapid Congo Red Removal
Source: Nanomaterials (Basel). 2024 Sep 17;14(18):1510. doi: 10.3390/nano14181510 (PMC11435052; doi:10.3390/nano14181510)
Supplement: Supplementary file 1 [file nanomaterials-14-01510-s001.zip › Supplementary Materials.pdf]

Assembly of Chitosan/Caragana Fiber to Construct Underwater Superelastic 2D layers-Supported 3D Architecture for Rapid Congo Red Removal

Ning Luo[1,†], Hanwen Ge[1,2,†], Xiangyu Liu[1], Qingdong He[1], Wenbo Wang[1,*], Wenyuan Ma[1], Fang Guo[1,*]

[1]College of Chemistry and Chemical Engineering, Inner Mongolia University, Hohhot 010021, China;

[2]SINOPEC Economic & Technical Research Institute Co., Ltd, Beijing 100029, China.

32307138@mail.imu.edu.cn (N.L.); ghw15001010907@163.com (H.G.); lxy13257244737@163.com (X.L.); 18847744604@163.com (Q.H.); wangwenbo@imu.edu.cn (W.W.); mawenyuaner@163.com (W.M.); guofang@imu.edu.cn (F.G.)

*Correspondence: wangwenbo@imu.edu.cn (W. W.); guofang@imu.edu.cn (F. G.)

†These authors contributed equally to this work.

1. Optimization of adsorbent

In order to obtain the optimal adsorption properties of the architecture material, the effects of adding amount of caragana fiber (0.05 g, 0.1 g, 0.15 g, 0.2 g, 0.25 g) on the adsorption capacity of the material were studied under other conditions unchanged (Fig. S4). As can be seen, with the increase of caragana fiber amount from 0.05 g to 0.25 g, the adsorption capacity of the architecture material first increased and then decreased. When the mass of caragana fiber is 0.20 g, the adsorption capacity of architecture material reached 140.30 mg/g, which proved that the CKF mass of 0.20 g is the optimal addition amount. The amount of CR adsorbed by the architecture material is the best. In the following experiments, the architecture material prepared under optimal reaction conditions was used to investigate the adsorption behavior, such as pH influence, adsorption performance through peristaltic pump, and removal efficiency at low concentration.

Supplementary Figures

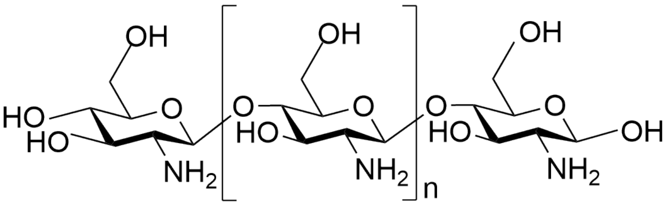

Fig. S1 The structure formula of chitosan [S1]

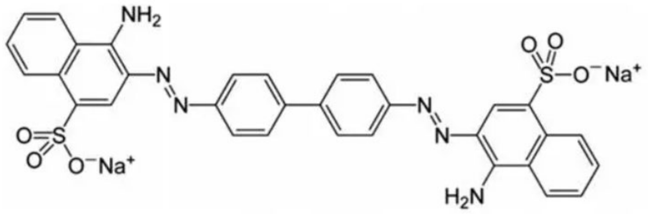

Fig. S2 The structure formula of Congo red.

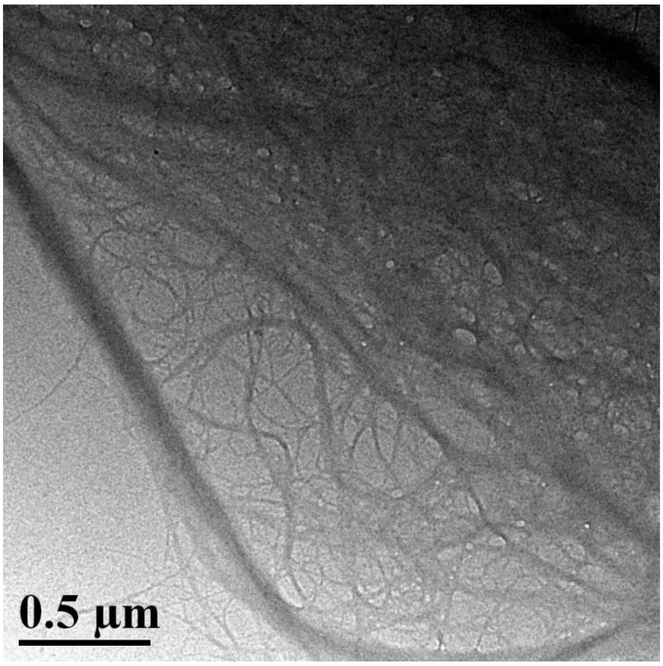

Fig. S3. TEM image of caragana fiber.

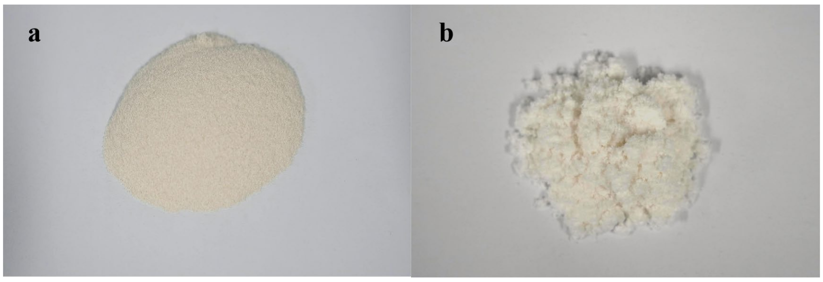

Fig. S4 Images of CS (a) and CKF (b).

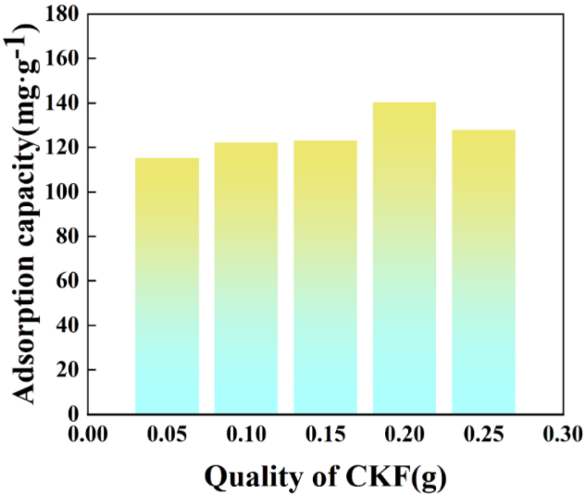

Fig. S5 Effect of CKF dosage in adsorbent on adsorption capacity.

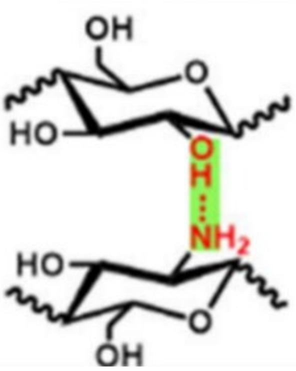

Fig. S6 Hydrogen bonding interaction between the hydroxyl group of CKF and the amino group of CS.

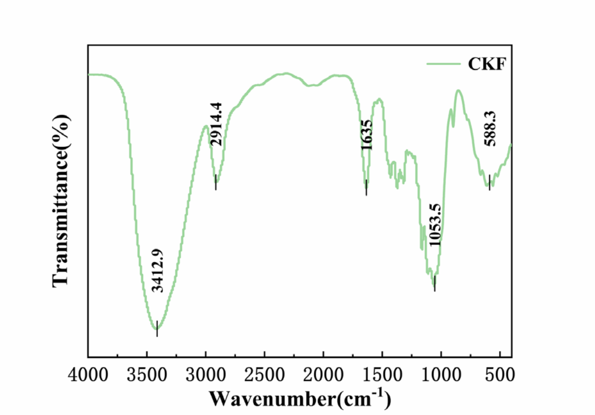

Fig. S7 FTIR spectrum of CKF.

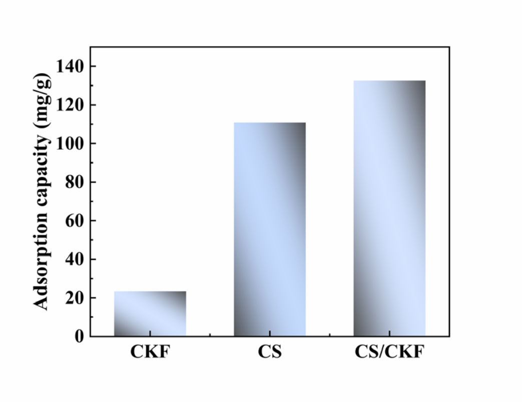

Fig. S8 Adsorption capacity of Congo red dye by CKF, CS and CKF/CS, respectively.

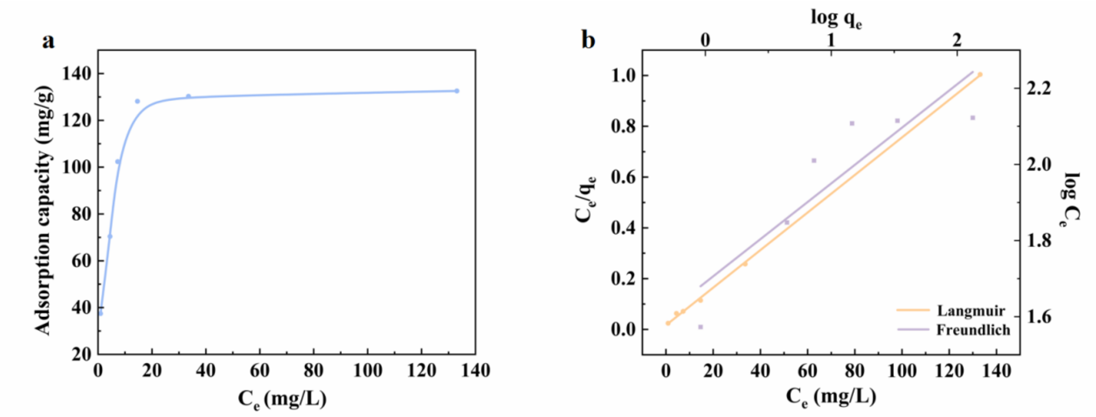

Fig. S9 (a) The curves of adsorption capacity versus equilibrium concentration (C_e, mg/L) for the adsorption of CR onto the CS/CKF composite material ; (b) The plots of C_e/q_e versus C_e for the adsorption of CR on the CS/CKF composite material (fitting with Langmuir model); and the plots of log q_e versus log C_e for the adsorption of CR on the CS/CKF composite material (fitting with Freundlich model).

Table S1 Fitting parameters with Langmuir model and Freundlich model for adsorption of CR

	Langmuir model			Freundlich model		
Samples	k_L(L/mg)	q_m(mg/g)	R^2	k_F (L/mg)$^{1/n}$	n	R^2
CS/CKF composite	0.446	135.12	0.9995	1.690	3.84	0.7929

Reference

[S1] Taghizadeh, M; Taghizadeh, A; Yazdi, M.K.; Zarrintaj, P.; Stadler, F.J.; Ramsey, J.D.; Habibzadeh, S.; Rad, S.H.; Naderi, G.; Saeb, M.R.; et al. Chitosan-based inks for 3D printing and bioprinting. Green Chem. **2022**, *24*, 62-101.
